# Pure yolk sac tumor of sacrococcygeal region

**DOI:** 10.4322/acr.2021.287

**Published:** 2021-05-25

**Authors:** Rashim Sharma, Sudeep Khera, Arvind Sinha, Taruna Yadav

**Affiliations:** 1 All India Institute of Medical Sciences, Department of Pathology and Lab Medicine, Jodhpur, Rajasthan, India; 2 All India Institute of Medical Sciences, Department of Pediatric Surgery, Jodhpur, Rajasthan, India; 3 All India Institute of Medical Sciences, Diagnostic and Interventional Radiology, Jodhpur, Rajasthan, India

**Keywords:** Child, Child, Preschool, Sacrococcygeal Region, Teratoma

## Abstract

The sacrococcygeal region is the most common site for the extragonadal germ cell tumors comprising seminomatous and non-seminomatous tumors. Seminomatous tumors are seminomas, and non-seminomatous tumors comprise mainly teratoma (mature and immature), yolk sac tumor (YST), embryonal carcinoma (EC), and choriocarcinoma. These tumors occur in newborns, infants, and adolescents. Other common sites for extragonadal germ cell tumors are the brain and mediastinum, although they may occur anywhere in the body. These tumors may occur in mixed as well as pure form. So, sectioning from different areas should be done before labeling them as pure germ cell tumors. YST, in its pure form, is rare and therefore should not be missed as it is chemosensitive. The patient should be thoroughly assessed clinically. Imaging also becomes necessary while evaluating swelling in the sacrococcygeal region and can aid in differentials. When the clinical and imaging suspicion of either Sacrococcygeal teratoma or other germ cell tumor is high, serum biomarkers as alfa-fetoprotein should be requested. The serum levels are necessary and should be done preoperatively, postoperatively, and during the course of chemotherapy as follow-up. However, the final diagnosis rests on the histopathological diagnosis. We report one such case of pure YST in the sacrococcygeal region in a 9-month-old female child. The imaging suggested sacrococcygeal teratoma type 4, and high alfa-fetoprotein levels were determined postoperatively.

## INTRODUCTION

YST is a rare neoplasm involving mostly the sacrococcygeal region. This entity must not be missed as it responds well to chemotherapy, which increases progression-free survival. YST may mimic Sacrococcygeal Teratoma (SCT) on imaging. Therefore, both entities should always be considered among the differential diagnosis. Here we discuss a case report of histopathologically diagnosed YST that mimicked Sacrococcygeal teratoma on imaging in a 9-month-old female child.

## CASE REPORT

**Figure 1:**
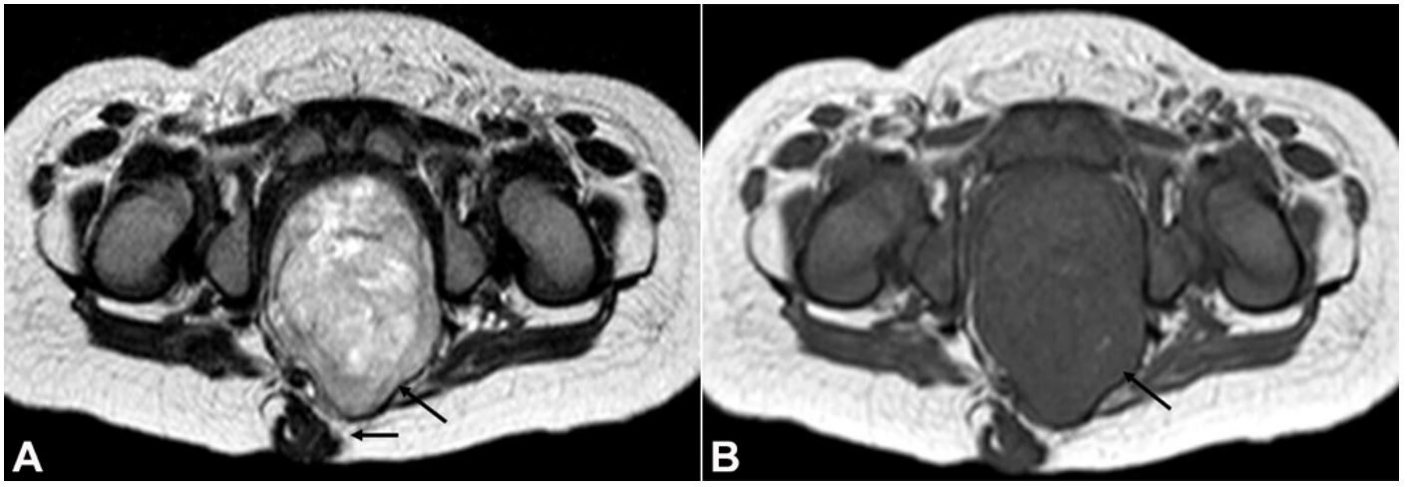
**A** – Axial T2 weighted image shows a heterogeneous hyperintense presacral mass (black arrows) filling the pelvic cavity reaching the gluteal cleft; **B** – Axial T1 weighted image shows a corresponding T1 hypointense mass (black arrow) with few punctate areas of hyperintensity.

**Figure 2:**
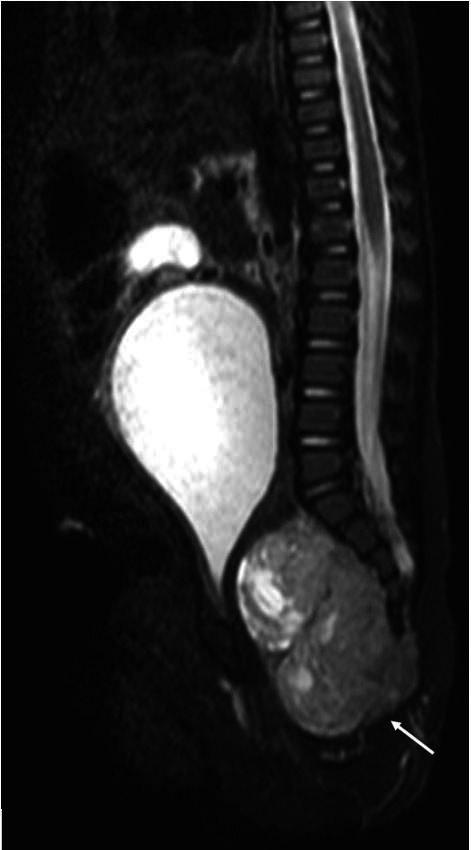
Sagittal STIR image showing the hyperintense mass (white arrow) anterior to the sacrococcygeal region compressing the rectum, anal canal, urinary bladder neck with overdistended urinary bladder extending to the mid-abdomen.

A 9-month-old female child was brought to the Out Patient Department (OPD) after noticing an increasing swelling in the lower back over the last 2 or 3 months. She, later on, developed constipation with a defecation frequency of once every 7 days. There was straining during micturition. No history of vomiting, limb weakness, fever, hematuria, shortness of breath was present. There was no family history of any malignancy. On clinical examination, no organomegaly was noted. A non-mobile, non-fluctuant and non-tender swelling was noted over the left side of the gluteal cleft. A magnetic resonance imaging (MRI) (Figure 1 and 2) revealed a large well-defined intrapelvic midline solid mass in the presacral space related to the coccyx measuring 4x3.6x6.6 cm with predominately soft tissue component with minimal cystic changes. The mass caused compression of the pelvic viscera. No evidence of any bone or adjacent organ invasion was present. On imaging, diagnosis of presacral purely intrapelvic sacrococcygeal teratoma type 4 was made with a small intraspinal extension.

**Figure 3:**
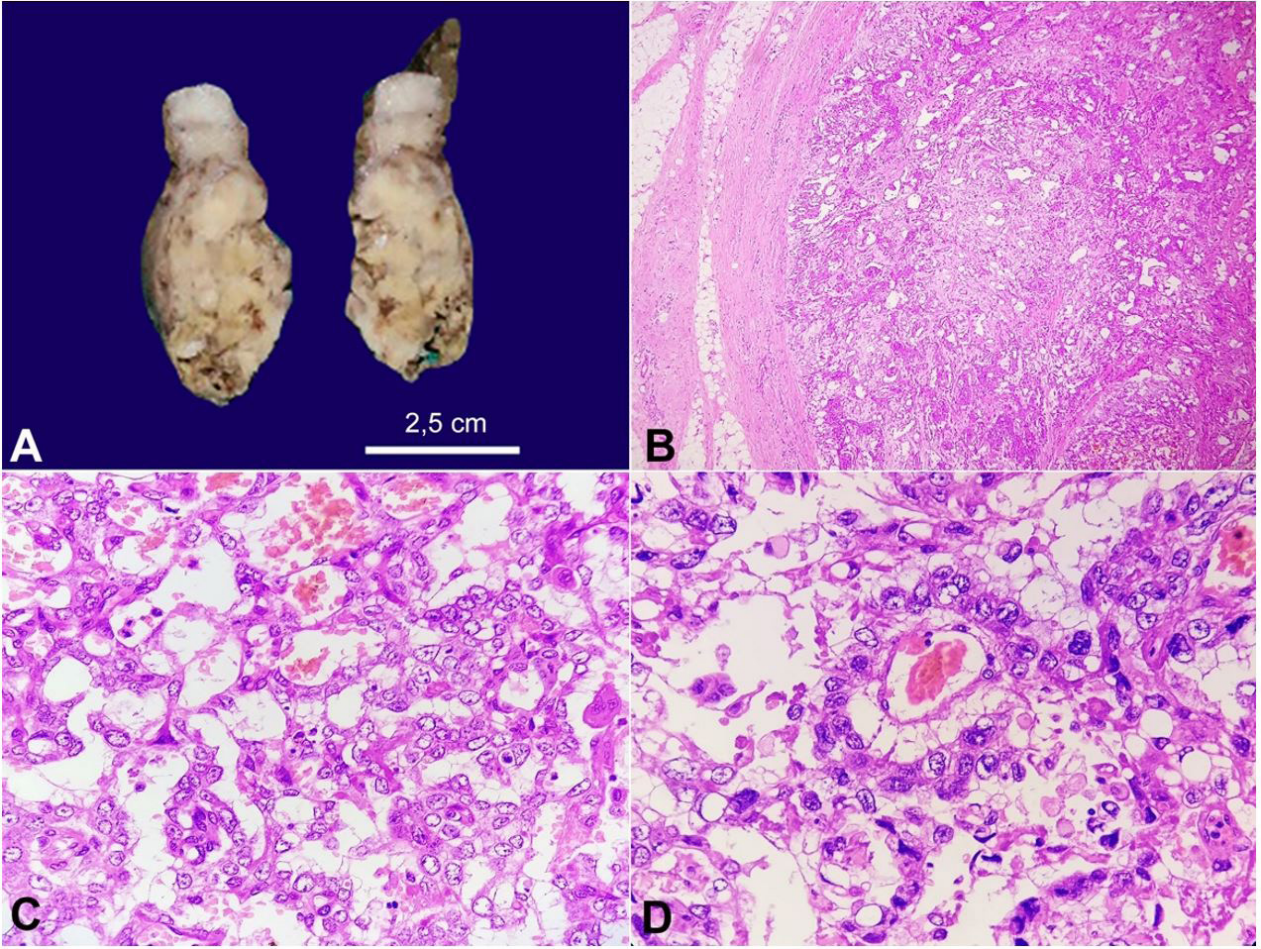
**A** – Gross view of the surgical specimen’s cut surface showing solid white areas with necrotic areas. **B**, **C**, and **D** – photomicrographs of the tumor; **B** – showing a tumor arranged in lobules with a fibrous capsule (4X, H&E); **C** – showing the microcystic pattern of the tumor cells (40X, H&E); **D** –Schiller Duval body (40X, H&E).

Tumor excision was performed, and the specimen was received with a skin-covered globular soft tissue mass measuring 7x5x3cm. The skin surface showed a nodule measuring 2x1cm. The cut surface showed a firm, solid white mass with areas showing necrosis and hemorrhage (Figure 3A). No hair, teeth, or a Rokitansky body were noted. The microscopy showed an invasive tumor arranged predominantly in a microcystic pattern, lobules, nests, papillae formation with a focal alveolar pattern, separated by fibrovascular septations (Figure 22C). Schiller Duval bodies were present (Figure. 2 D). Few bizarre cells with hyperchromatic nuclei and giant cells were also noted. Many eosinophilic globules were seen. Extensive necrosis areas, entrapped necrotic bony bits, focal cellular cartilaginous tissue, and numerous foamy histiocytes were also noted. The overlying skin was unremarkable.

**Figure 4:**
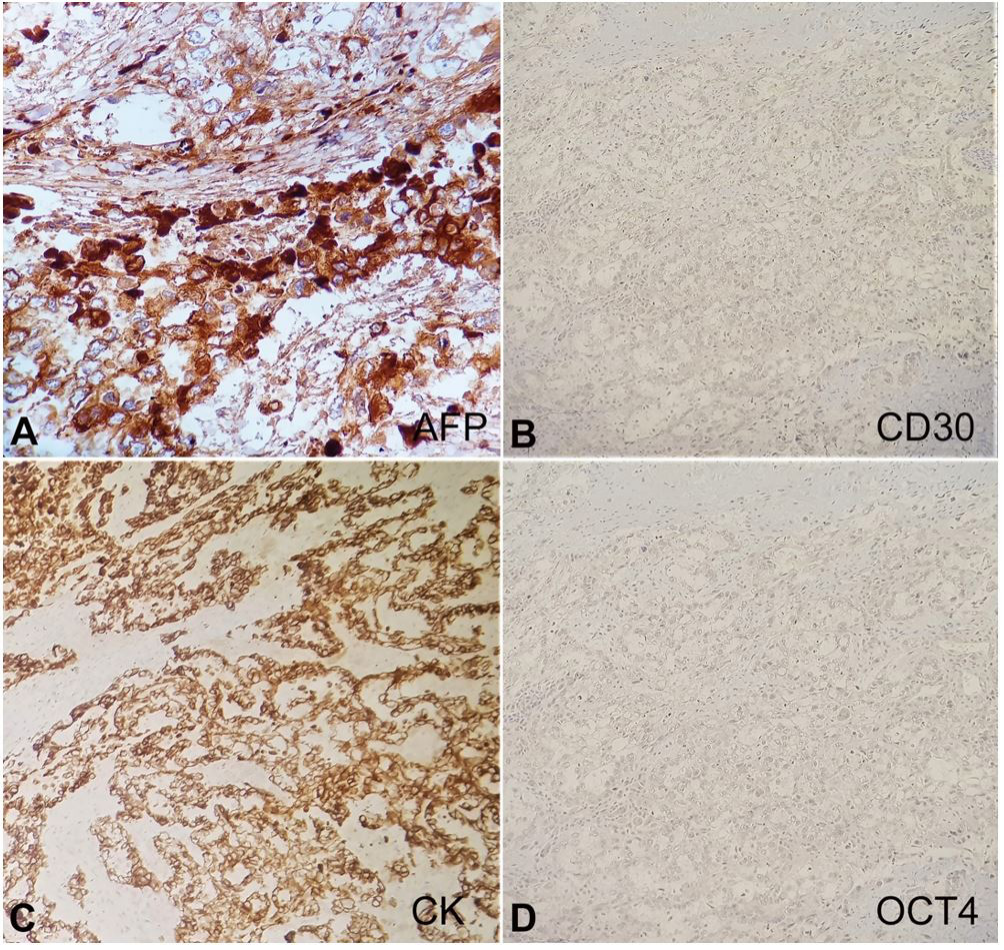
Immunohistochemical panel. **A** – AFP showing strong and diffuse positivity in the tumor cells; **B** – CD30 negative in tumor cells, **C** – showing strong and diffuse CK positivity in tumor cells; **D** – OCT4 negative in tumor cells.

On immunohistochemistry, the tumor cells were strong and diffusely positive for AFP (Figure 4A) and CK (cytokeratin) (Figure 4C) and negative for CD30, OCT4 (octamer binding transcription factor 4), and EMA (epithelial membrane antigen) (Figures 44D).

Alpha-fetoprotein (AFP) levels determined post-surgery were high (25000 IU). The final diagnosis rendered pure Yolk Sac Tumor. The patient has received 4 cycles of chemotherapy, BEP regimen (Bleomycin, Etoposide, and Cisplatin) and is still in follow-up. The subsequent AFP levels were 68.6 IU, 20.2 IU, and 8 IU.

## DISCUSSION

The sacrococcygeal region is the most common site for teratoma (mature and immature) occurring in newborns and infants, with an incidence between 1 per 35,000 to 1 per 50,000 live births.^1^ Sacrococcygeal teratoma (SCT) may be accompanied by another Germ Cell Tumor (GCT), mostly Yolk Sac Tumor (YST), but YST in its pure form is very rare. GCT accounts for 3% of all the neoplasms occurring in childhood, and among them, the YST is the most common type.^2^ As suggested by Teilum^3^, the primitive germ cell is the origin of the germ cell tumors, which give rise to dysgerminoma and tumors of totipotent cells. These totipotent cells have the potential for further differentiation. They either diversify into embryonal or somatic domain leading to teratoma or into extraembryonic domain forming either YST or choriocarcinoma.^3^ The Sacrococcygeal YST occurs commonly in children with age less than 3 years, and the most common presenting symptoms are constipation with swelling in the natal cleft area.^2^

The diagnosis of yolk sac tumors is challenging on imaging. However, a solid and cystic tumor with necrotic and hemorrhagic areas can give a clue to the diagnosis. They can look similar to sacrococcygeal teratomas, which show a soft tissue enhancement.^4^ The presence of bone or adjacent pelvic organ invasion or metastases indicates the malignant nature of the sacrococcygeal mass, which was absent in our case. Also, AFP levels may be elevated in both YST and other mixed GCT. ^5^

On the microscopy, several morphological patterns have been described. ^6^ These patterns are (i) microcystic, (ii) endodermal sinus, (iii) solid, (iv) myxomatous, (v) papillary, (vi) hepatoid, (vii) macrocystic, (vii) polyvesicular vitelline, (ix) glandular or (x) primitive endodermal and (xi) alveolar-glandular.^7^ Microcystic pattern is the most common pattern.^6^ ^,^ ^7^ The presence of Schiller Duval bodies and periodic acid positive (PAS) hyaline globules are distinctive findings in YST. However, Schiller Duval bodies are noted in only 50 to 75% of the cases. Thus, a thorough and extensive sampling is needed.

The recommended immunohistochemistry panel includes a general germ cell marker (SALL4), OCT4, AFP, and CD30. OCT 4 negativity negates the possibility of seminoma and embryonal tumor. CD30 negativity also rules out embryonal carcinoma. AFP is strong and diffusely granular in YST.

YST is chemo-responsive, with a survival rate of approximately 80%. ^8^ ^,^ ^9^ In locally advanced cases, the surgical resection is followed by chemotherapy comprising cisplatin-based regimens. After a meticulous search on pure YST in the sacrococcygeal region on PubMed and Cochrane, we found 4 case reports, 1 letter to the editor, 5 original articles, and 2 review articles, where a few cases of pure YST in the sacrococcygeal region has been mentioned.^2^ ^,^ ^10^ ^-^ ^20^ The data from various reports and original articles are shown in Table 1.^2^ ^,^ ^10^ ^-^ ^20^ Few studies showed that, prior to neoadjuvant chemotherapy (NACT), a mixed germ cell tumor was present, which after chemotherapy transformed to pure YST.^2^ ^,^ ^16^ ^,^ ^20^ Also, the determination of the AFP levels for diagnosis and therapeutic following up is needed in cases of YST.

**Table 1:** Various studies highlighting pure YST in chronological order

**authors**	# **of cases**	**Age range**	**“S” pure YST cases**	**Results**
Pedersen^10^	24	9m-36y	2	AFP was increased in all cases of endodermal sinus tumor or teratocarcinomas.
Ein^11^	15	11m-30m	10	2 out of 10 cases were malignant tumors
Hawkin^12^	89	1m-16y	15	No tumor behavior difference between “S” pure tumors and teratomas.
Davidof^2^	37	5m-16y	15	The overall, 2y survival rate was 60%. For YST of the “S” the survival rate was 70% post Chemo, AFP level monitoring was significant.
De Backer^13^	193	< 16Y	9	Patients with gonadal GCT had a higher overall survival than those with extragonadal GCT. Patients with cervical and mediastinal tumors had lower probability of event free survival than those with gonadal, retroperitoneal or sacrococcygeal GCT.
Wang et al.^14^	59	NA	27	All 59 YST cases were negative for OCT4, but strong positive for SALL4. More than 90% of the tumor area in 54 cases and 70% to 85% tumor area in 5 YST cases was noted making SALL 4 a novel and sensitive marker in YST. 66% cases showed focal PLAP staining.
Merchant and Stewart^15^	1	11m	1	Pure YST of sacrococcygeal region can be highlighted by other clinical features such as hypoechoic fluid collections in gluteal region.
Khanchel-Lakhoua et al.^16^	1	30m	1	Imaging, morphology and elevated AFP levels suggested YST of sacrococcygeal region.
Yoshid et al.^17^	289	<1m - >2y	NA	13 cases developed YST after resection of sacrococcygeal teratoma.
Pawar et al.^18^	1	2y	1	A clinical suspicion of YST should always be kept in infants with presentation at unusual locations.
Ben Nsir et al.^19^	1	18m	1	Pure YST can present with conus medullaris syndrome
Mondal and Mandal^20^	1	1.5m	1	GCT should always be suspected in young children presenting with lung metastases. AFP levels must be done while suspecting GCT.

m =month; GCT=germ cell tumor; “S” = sacrococcygeal region; Y= Year, YST= yolk sac tumor, AFP= alfa feto protein (Serum biomarker, elevated in YST and mixed GCT).

# =number, NA= Not applicable [Of 289 cases, 48 cases were mixed GCT].

In conclusion, YST should always be kept in the differential diagnosis when dealing with neoplasm of the sacrococcygeal region. AFP levels should be done before and after the surgical procedure, and the patient should be evaluated for a certain period of time on the basis of AFP levels. Also, YST can present with varied clinical scenarios in young patients and should not be overlooked, and suspicion of germ cell tumors should always be kept.

## References

[1] Gharpure V (2013). Sacrococcygeal teratoma. J Neonatal Surg.

[2] Davidoff AM, Hebra A, Bunin N, Shochat SJ, Schnaufer L (1996). Endodermal sinus tumor in children. J Pediatr Surg.

[3] Teilum G (1965). Classification of endodermal sinus tumour (mesoblatoma vitellinum) and so-called "embryonal carcinoma" of the ovary. Acta Pathol Microbiol Scand.

[4] Yoon HM, Byeon SJ, Hwang JY (2018). Sacrococcygeal teratomas in newborns: a comprehensive review for the radiologists. Acta Radiol.

[5] Talerman A, Haije WG, Baggerman L (1978). Serum alphafetoprotein (AFP) in diagnosis and management of endodermal sinus (yolk sac) tumor and mixed germ cell tumor of the ovary. Cancer.

[6] Teilum G (1976). Special tumors of ovary and testis and related extragonadal lesions: comparative pathology and histological indentification..

[7] Talerman A, Vang R, Kurman RJ, Ellenson LH, Ronnett BM (2011). Germ cell tumors of the ovary.. Blaustein's pathology of female genital tract.

[8] Papic JC, Finnell SM, Slaven JE, Billmire DF, Rescorla FJ, Leys CM (2014). Predictors of ovarian malignancy in children: overcoming clinical barriers of ovarian preservation. J Pediatr Surg.

[9] Billmire DF (2006). Germ cell tumors. Surgical Clinics..

[10] Norgaard‐Pedersen B, Albrechtsen R, Teilum G (1975). Serum alpha‐foetoprotein as a marker for endodermal sinus tumor (yolk sac tumor) or a vitelline component of ‘teratocarcinoma’. Acta Pathol Microbiol Scand [A].

[11] Ein SH, Mancer K, Adeyemi SD (1985). Malignant sacrococcygeal teratoma—endodermal sinus, yolk sac tumor—in infants and children: a 32-year review. J Pediatr Surg.

[12] Hawkins EP, Finegold MJ, Hawkins HK, Krischer JP, Starling KA, Weinberg A (1986). Nongerminomatous malignant germ cell tumors in children: a review of 89 cases from the Pediatric Oncology Group, 1971–1984. Cancer.

[13] De Backer A, Madern GC, Pieters R (2008). Influence of tumor site and histology on long-term survival in 193 children with extracranial germ cell tumors. Eur J Pediatr Surg.

[14] Wang F, Liu A, Peng Y (2009). Diagnostic utility of SALL4 in extragonadal yolk sac tumors: an immunohistochemical study of 59 cases with comparison to placental-like alkaline phosphatase, alpha-fetoprotein, and glypican-3. Am J Surg Pathol.

[15] Merchant A, Stewart RW (2010). Sacrococcygeal yolk sac tumor presenting as subcutaneous fluid collection initially treated as abscess. South Med J.

[16] Khanchel-Lakhoua F, Koubâa-Mahjoub W, Jouini R, Bel Haj Salah M, Kaabar N, Chadli-Debbiche A (2012). Sacrococcygeal yolk sac tumor: an uncommon site. APSP J Case Rep.

[17] Yoshida M, Matsuoka K, Nakazawa A (2013). Sacrococcygeal yolk sac tumor developing after teratoma: a clinicopathological study of pediatric sacrococcygeal germ cell tumors and a proposal of the pathogenesis of sacrococcygeal yolk sac tumors. J Pediatr Surg.

[18] Pawar NP, Mahajan SV, Chaudhari RA, Chavan SD (2013). Extragonadal GCT: A rare case report of sacrococcygeal pure yolk sac tumor. Indian J Pathol Microbiol.

[19] Ben Nsir A, Darmoul M, Arous SB, Hattab N (2015). Metastatic sacrococcygeal yolk sac tumor: a misleading diagnosis. J Neurosci Rural Pract.

[20] Mondal K, Mandal R (2017). Bilateral lung metastases unveils an asymptomatic sacrococcygeal yolk sac tumor. Indian J Pathol Microbiol.

